# Supplementary figures and images for: Screening of Lactiplantibacillus plantarum Strains from Sourdoughs for Biosuppression of Pseudomonas syringae pv. syringae and Botrytis cinerea in Table Grapes
Source: Microorganisms. 2022 Oct 22;10(11):2094. doi: 10.3390/microorganisms10112094 (PMC9696664; doi:10.3390/microorganisms10112094)

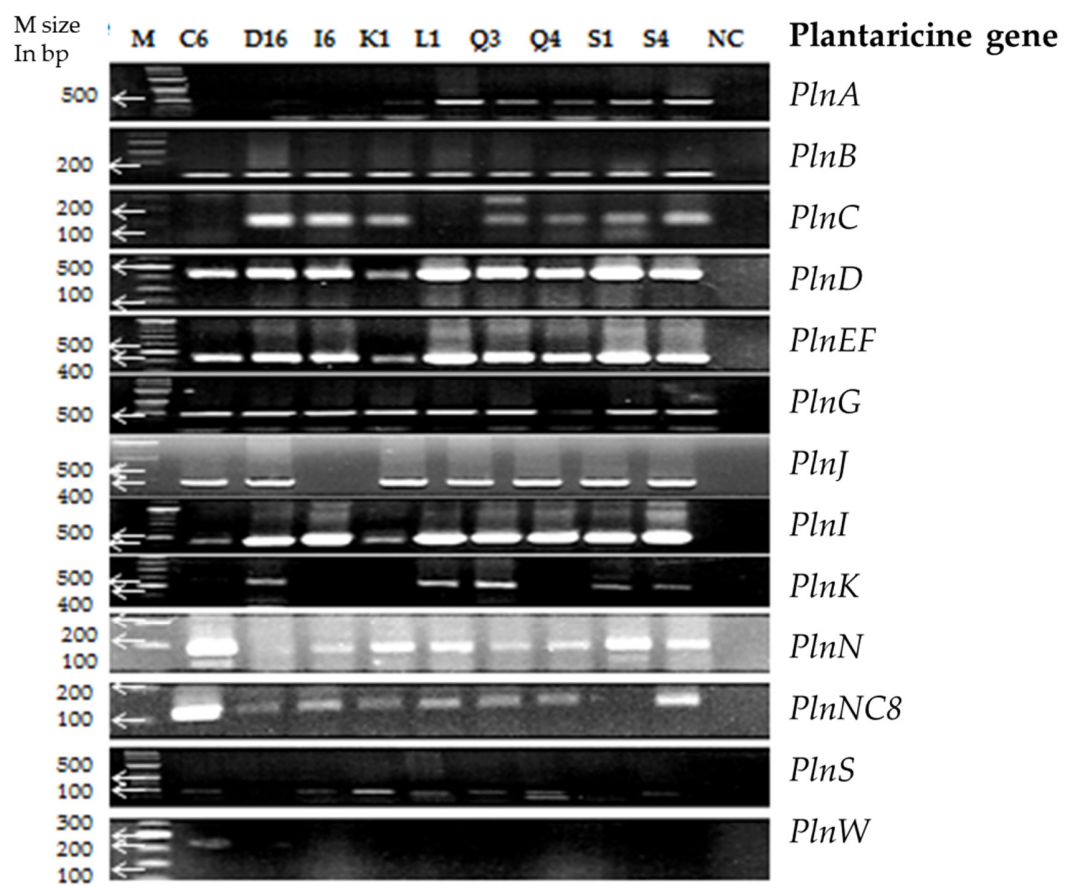

**Figure S1.** Plantaricin synthesis genes identified in LAB isolates.

Supplement: Supplementary file 1 [file microorganisms-10-02094-s001.zip › microorganisms-1953841 Supplementary.pdf]
